# Chiari Malformation and Attention Deficit Hyperactivity Disorder

**DOI:** 10.1155/2020/2694956

**Published:** 2020-09-22

**Authors:** Anaïs DuBow, Aurélie Mourot, Smadar Valérie Tourjman

**Affiliations:** ^1^Department of Psychiatry, University of Montreal, Montreal, QC, Canada; ^2^Department of Medicine, University of Montreal, Montreal, QC, Canada; ^3^Institut Universitaire en Santé Mentale de Montréal, Montreal, QC, Canada

## Abstract

Attention deficit hyperactivity disorder (ADHD) is a neurodevelopmental condition characterized by inattention, hyperactivity, and impulsivity. Chiari malformations (CM), first described approximately a hundred years ago, refer to a spectrum of hindbrain malformations characterized by cerebellar herniation through the foramen magnum. We present the case of a 28-year-old woman with ADHD and concurrent Chiari malformation type I (CM-I) that was diagnosed by CT scan. There is growing evidence supporting the role of the cerebellum and its associated structures in the pathophysiology of ADHD. Thus, a cerebellar malformation such as CM may impact neurological circuitry in a manner favoring the development of a neuropsychiatric disorder such as ADHD. Our case highlights the need for further studies pertaining to the role of the cerebellum in the pathophysiology of ADHD and the importance of considering the presence of CM when evaluating a patient with ADHD.

## 1. Introduction

Attention deficit hyperactivity disorder (ADHD) is a neurodevelopmental condition characterized by inattention, hyperactivity, and impulsivity [1]. Reported prevalence rates of ADHD vary in the literature. Causes for these differences include factors such as the population studied and the method of assessment [2, 3]. ADHD is a highly prevalent psychiatric disorder, affecting approximately 9% of children and 4% of adults in the United States, that emerges during childhood and persists into adulthood in as many as 75% of cases [4–6]. Despite recent advances in describing its precise pathophysiological mechanisms, much remains to be elucidated. Clinical observations of conditions which accompany and potentially contribute to ADHD may enhance our comprehension of this disorder.

Chiari malformations (CM), first described approximately a hundred years ago, refer to a spectrum of hindbrain malformations characterized by cerebellar herniation through the foramen magnum, resulting in compression of the caudal brainstem and upper cervical spinal cord as well as obstruction of normal cerebrospinal fluid (CSF) flow through the fourth ventricle. Possible CM aetiologies include genetic predisposition, congenital anomalies, and acquisition through trauma or illness [7]. Four types of CM (CM-I, CM-II, CM-III, and CM-IV) have been described and are classified according to cerebellar herniation, spinal cord spacing, and severity, with CM-I being the least severe form [7]. The symptoms most commonly encountered in CM-I are debilitating headaches that worsen with Valsalva maneuvers, neck pain, dizziness, and visual disturbances [8]. It is difficult to evaluate the true prevalence of CM; however, modern imaging techniques have enabled CM to be identified more frequently. Before these technologies were available CM, was evaluated to occur in .001% of births [9].

The case we describe here is one of only three reported individual cases described in the English literature of a co-occurrence of ADHD and CM-I.

## 2. Case Presentation

The patient is a 28-year-old Caucasian college student, who was referred by her primary care practitioner to our outpatient clinic in September 2018 for evaluation and treatment of ADHD.

She had been diagnosed with ADHD at age 12 by a pediatrician and a psychologist in the context of poor academic performance, concentration difficulties, attention deficits, distractibility, impulsivity, and lack of organizational skills. At that time, the patient almost failed her first year of high school and began a trial of psychostimulants. By age 28, she had tried several long-acting formulations of methylphenidate as well as a norepinephrine reuptake inhibitor, all of which increased her anxiety in social gatherings and resulted in significant dysphoria. At the time of her consultation, she was not on any medication.

She had no pertinent medical or surgical history, except for a concussion at age 15. Her mother had been diagnosed with depression and anxiety and her brother with borderline personality disorder.

One month prior to her consultation, the patient experienced a significant concussion accompanied by migraines, nausea, vomiting, and ataxia. An emergency physician prescribed a cerebral CT scan. While the results excluded hemorrhage, ischemia, or space-occupying lesions, they indicated a downward displacement of the cerebellar tonsils compatible with CM type I.

At the time of presentation to our clinic, most of her symptoms of concussion had subsided with the exception of dizziness during physical exercise. The patient was mainly preoccupied with symptoms of ADHD, which were evaluated with the clinical interview and the adult ADHD self-report scale (ASRS) [10]. Our consultation confirmed a postconcussion syndrome and ADHD. The patient's baseline ASRS score (before starting medication) was 62. She was concerned that her lifelong symptoms of ADHD may have been caused by the CM leading us to undertake a review of the literature.

In the months following her initial evaluation, the patient proved to be extremely sensitive to the side effects of stimulants, limiting the possibility of using the usual therapeutic dose range. She is currently stabilized on 10 mg of lisdexamfetamine dimesylate daily. At this dose, her ASRS score was 51. She notes some improvement in organisation but continues to have significant difficulties with concentration and impulsivity as of July 2019.

## 3. Discussion

Our case report of co-occurring ADHD and CM-I is one of only three cases described in the English literature. One other case occurred in a 10-year-old male who exhibited autism spectrum disorder (ASD), ADHD, and CM-I, and the third occurred in a 10-year-old male with a nonsense hemizygous mutation of the TBL1X gene, central congenital hypothyroidism, hearing loss, attention deficit and hyperactivity disorder (ADHD), autism spectrum disorder (ASD), encopresis, and CM-I [11, 12]. These reports are of interest as they support evidence pertaining to the role of the cerebellum, its genetics, and its associated structures in the pathophysiology of ADHD.

CM consists of the herniation of the cerebellar tonsils, through the foramen magnum resulting in alteration and compression of postero-inferior cerebellar structures. Cerebellar findings, notably smaller cerebellar volumes, are amongst some of the earliest reported differences noted in ADHD [13]. Variations in cerebellar structure and functional connectivity have been reported in ADHD and ADHD symptom severity has been shown to correlate with the degree of reduction in volume of both the posterior vermis and of the cerebellum as a whole [13, 14].

A review of the prevalence of psychiatric disorders in a sample of 86 children with a formal diagnosis of CM-I revealed elevated rates of psychiatric diagnoses, including ADHD (22.1%), anxiety (12.8%), and depression (10.5%) when compared to published norms in the general population [15].

Changes in the cerebellum and associated structures have also been linked with ASD, as was highlighted in the case report by Osuagwu and colleagues [11]. Their article underlined the neurobiological link between ADHD and ASD, as the cephalocranial disproportion in neural tissue in CM-I is also seen in ASD.

Furthermore, structural and functional imaging studies have shown evidence of altered brain volumes in ADHD leading to dysfunction in fronto-subcortical pathways [11]. A recent fMRI study noted that the altered connectivity in the cerebellar network was present in the combined type of ADHD (inattentive and hyperactive) as compared with alterations in the cingulo-frontoparietal attention and visual networks found in the predominately inattentive subtype [16].

Unfortunately, the absence of studies examining the association between ADHD and the different types of CM is a significant limitation of the available literature; it would be interesting to explore the relationship of severity of one condition and its correlation with that of the other. Additionally, an examination of the order of onset of cerebllar malformations and ADHD would inform the directionality of a causal relationship between the two conditions.

## 4. Conclusion

This case report suggests a potential link between CM and ADHD which may itself be, in part, a result of cerebellar dysfunction. Imaging might not be appropriate in every case of ADHD but may be considered when the clinical signs and symptoms of CM are present such as headaches, neck pain, dizziness, and visual disturbances. These observations add to data suggesting that the cerebellum and its associated neural networks may play a role in the pathophysiology of ADHD.

## Data Availability

Patient consent form and ASRS are available upon request.

## Abbreviations

ADHD: Attention deficit hyperactivity disorder
CM: Chiari malformation
ASD: Autism spectrum disorder
TBL1X: Transducin *β*-like 1 X-linked gene.
